# Supplementary material for: Genetic risk, adherence to healthy lifestyle and acute cardiovascular and thromboembolic complications following SARS-COV-2 infection
Source: Nat Commun. 2023 Aug 3;14:4659. doi: 10.1038/s41467-023-40310-0 (PMC10400557; doi:10.1038/s41467-023-40310-0)
Supplement: Supplementary file 1 — Supplementary Information [file 41467_2023_40310_MOESM1_ESM.pdf]

Supplementary Results

sTable 1: Characteristics of participants with COVID-19 overall and in subgroups by the genetic risk of coronary artery disease

Participant characteristics	All eligible participants[1]	Participants with COVID-19				
		All	Low genetic risk	Intermediate genetic risk	High genetic risk	SMD
Number	407453	106005	21201	63603	21201	
Mean age, year (SD)	69.46 (8.14)	67.68 (8.26)	67.48 (8.27)	67.72 (8.25)	67.77 (8.25)	0.023
sex, No. (%)						
Female	225700 (55.4)	58045 (54.8)	11559 (54.5)	34930 (54.9)	11556 (54.5)	0.006
Male	181752 (44.6)	47960 (45.2)	9642 (45.5)	28673 (45.1)	9645 (45.5)	
Ethnicity, No. (%)						
White	375824 (92.2)	94821 (89.4)	18755 (88.5)	57059 (89.7)	19007 (89.7)	0.027
Other ethnic groups	31629 (7.8)	11184 (10.6)	2446 (11.5)	6544 (10.3)	2194 (10.3)	
Obesity, No. (%)						
BMI < 30	310852 (76.3)	80339 (75.8)	15700 (74.1)	48282 (75.9)	16357 (77.2)	0.048
BMI ≥ 30	96601 (23.7)	25666 (24.2)	5501 (25.9)	15321 (24.1)	4844 (22.8)	
Socioeconomic deprivation[2], mean (SD)	17.47 (13.75)	17.13 (13.19)	17.65 (13.52)	17.04 (13.07)	16.86 (13.21)	0.04
Education categories[3], No. (%)						
I	72698 (17.8)	13808 (13.0)	3099 (14.6)	8235 (12.9)	2474 (11.7)	0.082
II	111099 (27.3)	30324 (28.6)	6270 (29.6)	18301 (28.8)	5753 (27.1)	
III	45501 (11.2)	12762 (12.0)	2514 (11.9)	7592 (11.9)	2656 (12.5)	
IV	20685 (5.1)	5113 (4.8)	1027 (4.8)	3080 (4.8)	1006 (4.7)	
V	157469 (38.6)	43998 (41.5)	8291 (39.1)	26395 (41.5)	9312 (43.9)	
**Individual lifestyle factors, No. (%)**						
Smoking status						
Never or past	367963 (90.3)	96827 (91.3)	19294 (91.0)	58074 (91.3)	19459 (91.8)	0.019
Current	39490 (9.7)	9178 (8.7)	1907 (9.0)	5529 (8.7)	1742 (8.2)	
Alcohol intake						
≤ 4 times week	324878 (79.7)	85137 (80.3)	17212 (81.2)	51072 (80.3)	16853 (79.5)	0.028
Daily or almost daily	82575 (20.3)	20868 (19.7)	3989 (18.8)	12531 (19.7)	4348 (20.5)	
Physical activity						
≥150 min per week of MIPA or ≥75 min per week of VIPA	344442 (84.5)	88608 (83.6)	17657 (83.3)	53218 (83.7)	17733 (83.6)	0.007

<150 min per week of MIPA & <75 min per week of VIPA	63011 (15.5)	17397 (16.4)	3544 (16.7)	10385 (16.3)	3468 (16.4)	
Television viewing time						
< 4 h/day	293745 (72.1)	79656 (75.1)	15548 (73.3)	47827 (75.2)	16281 (76.8)	0.053
≥ 4 h/day	113708 (27.9)	26349 (24.9)	5653 (26.7)	15776 (24.8)	4920 (23.2)	
Sleep duration						
≥7 & ≤9h/day	301120 (73.9)	79162 (74.7)	15616 (73.7)	47554 (74.8)	15992 (75.4)	0.027
<7 or >9h/day	106333 (26.1)	26843 (25.3)	5585 (26.3)	16049 (25.2)	5209 (24.6)	
Fruit and vegetable intake						
≥ 400 g/day	326692 (80.2)	84622 (79.8)	16878 (79.6)	50750 (79.8)	16994 (80.2)	0.009
<400 g/ day	80761 (19.8)	21383 (20.2)	4323 (20.4)	12853 (20.2)	4207 (19.8)	
Oily fish intake						
≥1 portion/week	229373 (56.3)	56737 (53.5)	11354 (53.6)	33965 (53.4)	11418 (53.9)	0.006
<1 portion/week	178080 (43.7)	49268 (46.5)	9847 (46.4)	29638 (46.6)	9783 (46.1)	
Red meat intake						
≤3 portion/week	187802 (46.1)	49840 (47.0)	10307 (48.6)	29856 (46.9)	9677 (45.6)	0.04
>3 portion/week	219651 (53.9)	56165 (53.0)	10894 (51.4)	33747 (53.1)	11524 (54.4)	
Processed meat intake						
≤1 portion/week	281938 (69.2)	72015 (67.9)	14542 (68.6)	43087 (67.7)	14386 (67.9)	0.012
>1 portion/week	125515 (30.8)	33990 (32.1)	6659 (31.4)	20516 (32.3)	6815 (32.1)	
Composite healthy lifestyle, No. (%)						
Unfavourable	30746 (7.5)	7816 (7.4)	1558 (7.3)	4760 (7.5)	1498 (7.1)	0.013
Moderate	154864 (38.0)	40238 (38.0)	8101 (38.2)	24101 (37.9)	8036 (37.9)	
Favourable	221843 (54.4)	57951 (54.7)	11542 (54.4)	34742 (54.6)	11667 (55.0)	

BMI: Body mass index. MIPA: Moderate intensity physical activity. VIPA: vigorous intensity physical activity. SMD: standardized mean difference

[1] All eligible participants in UK Biobank included those who survived when this study began (March 1, 2020).[2] High score indicates higher levels of deprivation. [3] Education category I includes self-reported "None of the above" and "Prefer not to answer", II includes "CSEs or equivalent" and "O levels/GCSEs or equivalent", III includes "A levels/AS levels or equivalent", IV includes "Other professional qualifications e.g.: nursing, teaching", and V includes "NVQ or HND or HNC or equivalent" and "College or University degree."

sTable 2: Characteristics of participants with COVID-19 overall and in subgroups by the genetic risk of venous thromboembolism

Participant characteristics	All eligible participants[2]	Participants with COVID-19				
		All	Low genetic risk	Intermediate genetic risk	High genetic risk	SMD
Number	407453	106005	21201	63603	21201	
Mean age, year (SD)	69.46 (8.14)	67.68 (8.26)	67.59 (8.28)	67.73 (8.23)	67.63 (8.30)	0.012
sex, No. (%)						
Female	225700 (55.4)	58045 (54.8)	11573 (54.6)	34805 (54.7)	11667 (55.0)	0.006
Male	181752 (44.6)	47960 (45.2)	9628 (45.4)	28798 (45.3)	9534 (45.0)	
Ethnicity, No. (%)						
White	375824 (92.2)	94821 (89.4)	18868 (89.0)	57009 (89.6)	18944 (89.4)	0.014
Other ethnic groups	31629 (7.8)	11184 (10.6)	2333 (11.0)	6594 (10.4)	2257 (10.6)	
Obesity, No. (%)						
BMI < 30	310852 (76.3)	80339 (75.8)	15918 (75.1)	48075 (75.6)	16346 (77.1)	0.032
BMI ≥ 30	96601 (23.7)	25666 (24.2)	5283 (24.9)	15528 (24.4)	4855 (22.9)	
Socioeconomic deprivation, mean (SD)	17.47 (13.75)	17.13 (13.19)	17.11 (13.19)	17.15 (13.22)	17.08 (13.11)	0.004
Education categories[3], No. (%)						
I	72698 (17.8)	13808 (13.0)	2764 (13.0)	8372 (13.2)	2672 (12.6)	0.023
II	111099 (27.3)	30324 (28.6)	6077 (28.7)	18316 (28.8)	5931 (28.0)	
III	45501 (11.2)	12762 (12.0)	2612 (12.3)	7551 (11.9)	2599 (12.3)	
IV	20685 (5.1)	5113 (4.8)	1018 (4.8)	3085 (4.9)	1010 (4.8)	
V	157469 (38.6)	43998 (41.5)	8730 (41.2)	26279 (41.3)	8989 (42.4)	
**Individual lifestyle factors, No. (%)**						
Smoking status						
Never or past	367963 (90.3)	96827 (91.3)	19381 (91.4)	58027 (91.2)	19419 (91.6)	0.009
Current	39490 (9.7)	9178 (8.7)	1820 (8.6)	5576 (8.8)	1782 (8.4)	
Alcohol intake						
≤ 4 times week	324878 (79.7)	85137 (80.3)	17080 (80.6)	51035 (80.2)	17022 (80.3)	0.005
Daily or almost daily	82575 (20.3)	20868 (19.7)	4121 (19.4)	12568 (19.8)	4179 (19.7)	
Physical activity						
≥150 min per week of MIPA or ≥75 min per week of VIPA	344442 (84.5)	88608 (83.6)	17662 (83.3)	53155 (83.6)	17791 (83.9)	0.011
<150 min per week of MIPA & <75 min per week of VIPA	63011 (15.5)	17397 (16.4)	3539 (16.7)	10448 (16.4)	3410 (16.1)	
Television viewing time						

< 4 h/day	293745 (72.1)	79656 (75.1)	15911 (75.0)	47693 (75.0)	16052 (75.7)	0.011
≥ 4 h/day	113708 (27.9)	26349 (24.9)	5290 (25.0)	15910 (25.0)	5149 (24.3)	
Sleep duration						
≥7 & ≤9h/day	301120 (73.9)	79162 (74.7)	15744 (74.3)	47551 (74.8)	15867 (74.8)	0.009
<7 or >9h/day	106333 (26.1)	26843 (25.3)	5457 (25.7)	16052 (25.2)	5334 (25.2)	
Fruit and vegetable intake						
≥ 400 g/day	326692 (80.2)	84622 (79.8)	17030 (80.3)	50654 (79.6)	16938 (79.9)	0.011
<400 g/ day	80761 (19.8)	21383 (20.2)	4171 (19.7)	12949 (20.4)	4263 (20.1)	
Oily fish intake						
≥1 portion/week	229373 (56.3)	56737 (53.5)	11380 (53.7)	34009 (53.5)	11348 (53.5)	0.003
<1 portion/week	178080 (43.7)	49268 (46.5)	9821 (46.3)	29594 (46.5)	9853 (46.5)	
Red meat intake						
≤3 portion/week	187802 (46.1)	49840 (47.0)	9912 (46.8)	29989 (47.2)	9939 (46.9)	0.005
>3 portion/week	219651 (53.9)	56165 (53.0)	11289 (53.2)	33614 (52.8)	11262 (53.1)	
Processed meat intake						
≤1 portion/week	281938 (69.2)	72015 (67.9)	14427 (68.0)	43137 (67.8)	14451 (68.2)	0.005
>1 portion/week	125515 (30.8)	33990 (32.1)	6774 (32.0)	20466 (32.2)	6750 (31.8)	
Composite healthy lifestyle, No. (%)						
Unfavourable	30746 (7.5)	7816 (7.4)	1569 (7.4)	4770 (7.5)	1477 (7.0)	0.014
Moderate	154864 (38.0)	40238 (38.0)	8069 (38.1)	24120 (37.9)	8049 (38.0)	
Favourable	221843 (54.4)	57951 (54.7)	11563 (54.5)	34713 (54.6)	11675 (55.1)	

BMI: Body mass index. MIPA: Moderate intensity physical activity. VIPA: vigorous intensity physical activity. SMD: standardized mean difference

[1] All eligible participants in UK Biobank included those who survived when this study began (March 1, 2020).[2] High score indicates higher levels of deprivation. [3] Education category I includes self-reported “None of the above” and “Prefer not to answer”, II includes “CSEs or equivalent” and “O levels/GCSEs or equivalent”, III includes “A levels/AS levels or equivalent”, IV includes “Other professional qualifications e.g.: nursing, teaching”, and V includes “NVQ or HND or HNC or equivalent” and “College or University degree.”

sTable 3: Characteristics of participants with COVID-19 overall and in subgroups by the genetic risk of ischaemic stroke

Participant characteristics	All eligible participants[2]	Participants with COVID-19				
		All	Low genetic risk	Intermediate genetic risk	High genetic risk	SMD
Number	407453	106005	21201	63603	21201	
Mean age, year (SD)	69.46 (8.14)	67.68 (8.26)	67.55 (8.24)	67.69 (8.27)	67.79 (8.22)	0.02
sex, No. (%)						
Female	225700 (55.4)	58045 (54.8)	11545 (54.5)	34878 (54.8)	11622 (54.8)	0.005
Male	181752 (44.6)	47960 (45.2)	9656 (45.5)	28725 (45.2)	9579 (45.2)	
Ethnicity, No. (%)						
White	375824 (92.2)	94821 (89.4)	18976 (89.5)	57108 (89.8)	18737 (88.4)	0.03
Other ethnic groups	31629 (7.8)	11184 (10.6)	2225 (10.5)	6495 (10.2)	2464 (11.6)	
Obesity, No. (%)						
BMI < 30	310852 (76.3)	80339 (75.8)	15125 (71.3)	48316 (76.0)	16898 (79.7)	0.13
BMI ≥ 30	96601 (23.7)	25666 (24.2)	6076 (28.7)	15287 (24.0)	4303 (20.3)	
Socioeconomic deprivation, mean (SD)	17.47 (13.75)	17.13 (13.19)	17.77 (13.64)	17.06 (13.12)	16.68 (12.94)	0.055
Education categories[3], No. (%)						
I	72698 (17.8)	13808 (13.0)	3188 (15.0)	8276 (13.0)	2344 (11.1)	0.108
II	111099 (27.3)	30324 (28.6)	6303 (29.7)	18303 (28.8)	5718 (27.0)	
III	45501 (11.2)	12762 (12.0)	2534 (12.0)	7602 (12.0)	2626 (12.4)	
IV	20685 (5.1)	5113 (4.8)	1067 (5.0)	3061 (4.8)	985 (4.6)	
V	157469 (38.6)	43998 (41.5)	8109 (38.2)	26361 (41.4)	9528 (44.9)	
**Individual lifestyle factors, No. (%)**						
Smoking status						
Never or past	367963 (90.3)	96827 (91.3)	19193 (90.5)	58109 (91.4)	19525 (92.1)	0.037
Current	39490 (9.7)	9178 (8.7)	2008 (9.5)	5494 (8.6)	1676 (7.9)	
Alcohol intake						
≤ 4 times week	324878 (79.7)	85137 (80.3)	17223 (81.2)	50980 (80.2)	16934 (79.9)	0.023
Daily or almost daily	82575 (20.3)	20868 (19.7)	3978 (18.8)	12623 (19.8)	4267 (20.1)	
Physical activity						
≥150 min per week of MIPA or ≥75 min per week of VIPA	344442 (84.5)	88608 (83.6)	17546 (82.8)	53226 (83.7)	17836 (84.1)	0.025
<150 min per week of MIPA & <75 min per week of VIPA	63011 (15.5)	17397 (16.4)	3655 (17.2)	10377 (16.3)	3365 (15.9)	
Television viewing time						

< 4 h/day	293745 (72.1)	79656 (75.1)	15413 (72.7)	47719 (75.0)	16524 (77.9)	0.081
≥ 4 h/day	113708 (27.9)	26349 (24.9)	5788 (27.3)	15884 (25.0)	4677 (22.1)	
Sleep duration						
≥7 & ≤9h/day	301120 (73.9)	79162 (74.7)	15487 (73.0)	47614 (74.9)	16061 (75.8)	0.041
<7 or >9h/day	106333 (26.1)	26843 (25.3)	5714 (27.0)	15989 (25.1)	5140 (24.2)	
Fruit and vegetable intake						
≥ 400 g/day	326692 (80.2)	84622 (79.8)	16710 (78.8)	50852 (80.0)	17060 (80.5)	0.027
<400 g/ day	80761 (19.8)	21383 (20.2)	4491 (21.2)	12751 (20.0)	4141 (19.5)	
Oily fish intake						
≥1 portion/week	229373 (56.3)	56737 (53.5)	11154 (52.6)	33994 (53.4)	11589 (54.7)	0.027
<1 portion/week	178080 (43.7)	49268 (46.5)	10047 (47.4)	29609 (46.6)	9612 (45.3)	
Red meat intake						
≤3 portion/week	187802 (46.1)	49840 (47.0)	10405 (49.1)	29919 (47.0)	9516 (44.9)	0.056
>3 portion/week	219651 (53.9)	56165 (53.0)	10796 (50.9)	33684 (53.0)	11685 (55.1)	
Processed meat intake						
≤1 portion/week	281938 (69.2)	72015 (67.9)	14308 (67.5)	43226 (68.0)	14481 (68.3)	0.012
>1 portion/week	125515 (30.8)	33990 (32.1)	6893 (32.5)	20377 (32.0)	6720 (31.7)	
Composite healthy lifestyle, No. (%)						
Unfavourable	30746 (7.5)	7816 (7.4)	1700 (8.0)	4629 (7.3)	1487 (7.0)	0.041
Moderate	154864 (38.0)	40238 (38.0)	8224 (38.8)	24168 (38.0)	7846 (37.0)	
Favourable	221843 (54.4)	57951 (54.7)	11277 (53.2)	34806 (54.7)	11868 (56.0)	

BMI: Body mass index. MIPA: Moderate intensity physical activity. VIPA: vigorous intensity physical activity. SMD: standardized mean difference

[1] All eligible participants in UK Biobank included those who survived when this study began (March 1, 2020).[2] High score indicates higher levels of deprivation. [3] Education category I includes self-reported “None of the above” and “Prefer not to answer”, II includes “CSEs or equivalent” and “O levels/GCSEs or equivalent”, III includes “A levels/AS levels or equivalent”, IV includes “Other professional qualifications e.g.: nursing, teaching”, and V includes “NVQ or HND or HNC or equivalent” and “College or University degree.”

sTable 4: Summary statistics of cardiovascular and thromboembolic events following COVID-19

Outcomes	Total follow-up (days)	Number of events	Incidence rate per 1,000 person-years	Cumulative incidence (%)
Atrial fibrillation	9097074	1397	56.05	1.31
Coronary artery disease	9129955	733	29.30	0.69
Venous thromboembolism	9152751	244	9.73	0.23
Ischemic stroke	9161711	104	4.14	0.09

sFigure 1: Distribution of the polygenic risk scores and test of potential non-linear relationships.

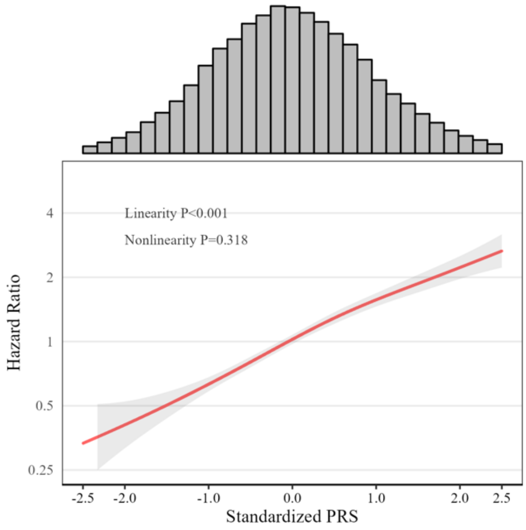

Atrial fibrillation

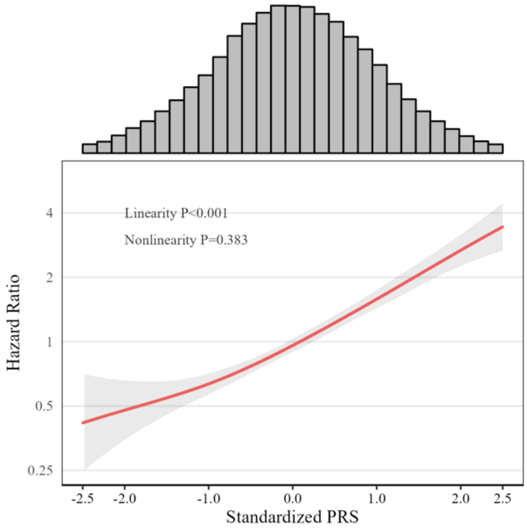

Coronary artery disease

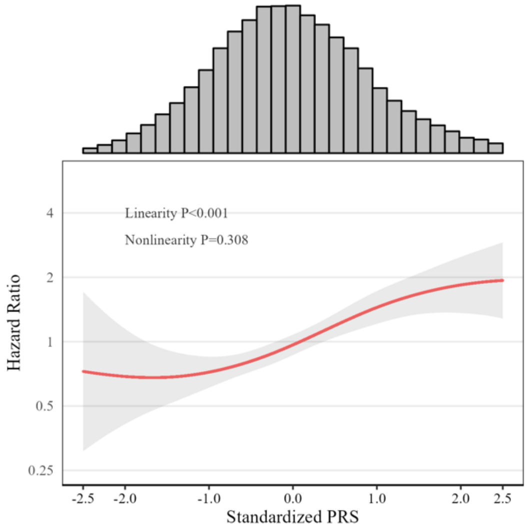

Venous thromboembolism

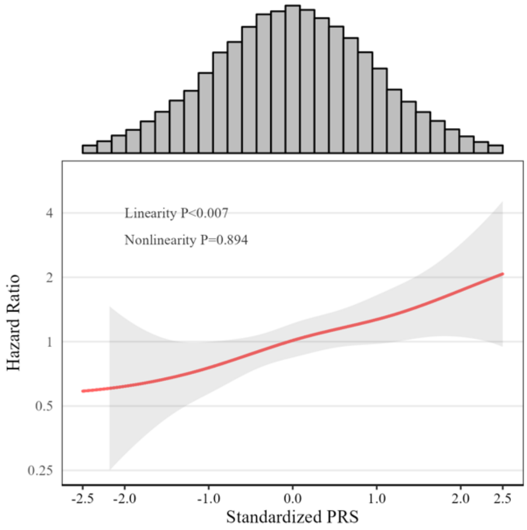

Ischemic stroke

The confidence interval of hazard ratios was removed if it was larger than 4 or smaller than 0.25. The penalized splines for the Cox proportional hazards model were implemented using the "pspline" function in R, with the degrees of freedom (df) set at 3. Two p-values based on likelihood ratio test statistics with a chi-square distribution are reported: one evaluates the significance of the linear term and the other evaluates the significance of the non-linear terms. A significant p-value for the non-linear terms indicates that the relationship between the polygenic risk score and the hazard is not linear..

sFigure 2: Adjusted hazard ratios of each healthy lifestyle factor

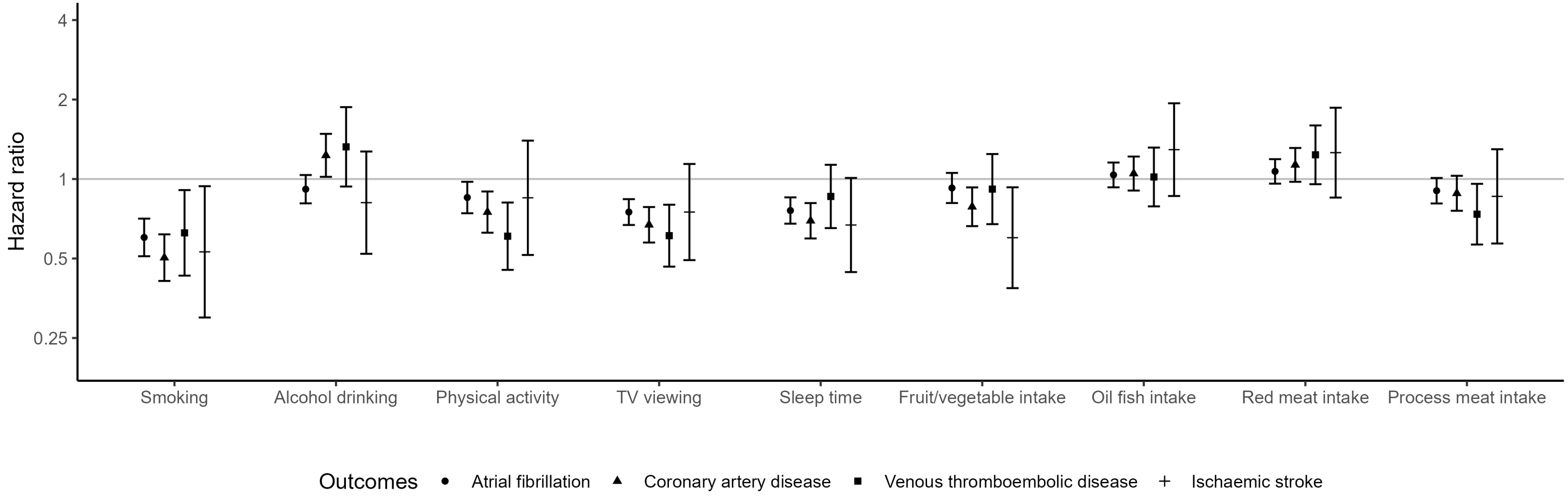

Point estimates are provided with 95% confidence intervals without correcting for multiple testing.

sTable 5: Adjusted hazard ratios in sensitivity analyses

	Atrial fibrillation	Coronary artery disease	Venous thromboembolism	Ischemic stroke
**Sensitivity analysis for the genetic exposure**				
Main results	1.52 (1.44 to 1.60)	1.57 (1.46 to 1.69)	1.33 (1.18 to 1.50)	1.27 (1.05 to 1.55)
Use of enhanced PRS	1.58 (1.40 to 1.77)	1.68 (1.45 to 1.95)	1.38 (1.09 to 1.76)	1.40 (0.91 to 2.15)
Incident CVE events[1]	1.47 (1.37 to 1.59)	1.69 (1.53 to 1.88)	1.35 (1.19 to 1.53)	1.25 (1.01 to 1.54)
Primary hospital admission diagnoses[2]	1.50 (1.37 to 1.64)	1.57 (1.39 to 1.78)	1.27 (1.08 to 1.48)	0.94 (0.65 to 1.35)
Diabetes (Negative control outcome)[3]	0.91 (0.81 to 1.02	1.06 (0.94 to 1.18)	1.11 (0.98 to 1.24)	1.07 (0.96 to 1.20)
**Sensitivity analysis for the lifestyle exposure**				
Main results	0.88 (0.85 to 0.92)	0.85 (0.81 to 0.90)	0.87 (0.79 to 0.95)	0.84 (0.73 to 0.97)
Incident cardiovascular and thromboembolic events[1]	0.90 (0.85 to 0.95)	0.87 (0.81 to 0.94)	0.87 (0.79 to 0.96)	0.90 (0.77 to 1.05)
Primary hospital admission diagnoses[2]	0.87 (0.81 to 0.93)	0.84 (0.77 to 0.92)	0.90 (0.80 to 1.01)	0.76 (0.59 to 0.98)

[1]Number of incident events were 677, 365, 225 and 90, respectively, for atrial fibrillation, coronary artery disease, venous thromboembolism and Ischaemic stroke. [2]Number of events related hospital admission were 442, 248, 141 and 29, accordingly. [3]Number of diabetes event was 289 during the same 90 days' follow-up as the primary outcomes.

Supplementary Methods

UK Biobank self-reported highest qualification mapped to the International Standard Classification of Education (ISCED).

UK Biobank self-reported highest qualification	Years of education	ISCED
Prefer not to answer	7	1
None of the above	7	1
CSEs or equivalent	10	2
O levels/GCSEs or equivalent	10	2
A levels/AS levels or equivalent	13	3
Other professional qualifications eg: nursing, teaching	15	4
NVQ or HND or HNC or equivalent	19	5
College or University degree	20	5

Variables used to create the lifestyle score.

Lifestyle factor	Questionnaire	Healthy (0)	Unhealthy (1)
**1. Smoking status**	"Do you smoke tobacco now?" and "In the past, how often have you smoked tobacco?"	past or never smoker	current
**2. Alcohol intake**	"About how often do you drink alcohol?"	≤ 4 times week	Daily or almost daily
**3. Physical activity**	IPAQ short form2– total time walking or moderate and vigorous-intensity PA in previous week *	≥150 min/week moderate or ≥ 75 min/week vigorous PA	< 75 min/week vigorous PA
**4. TV viewing /sedentary time**	"In a typical day, how many hours do you spend watching TV?" ǂ	< 4 h/day	≥ 4 h/day
**5. Sleep time**	"About how many hours sleep do you get in every 24 hours?"	>7 or <9h/day	<7 or >9h/day
**Dietary characteristics**	Individual dietary components contributed directly to lifestyle score.ф		
**6. Fruit and vegetable intake**	"About how many of …. would you eat per day?" Separate questions for pieces of fresh and dried fruit, tablespoons of salad or cooked/raw vegetables. Combined and converted to g/day (1 portion = 80 g)	≥ 400 g/day	<400 g/ day
**7. Oily fish intake**	"How often do you eat oily fish? (e.g. sardines, salmon, mackerel, herring)"	≥1 portion/week	<1 portion/week
**8. Red meat intake**	"How often do you eat…?" Separate questions for Beef / lamb or mutton / pork (excluding processed meats such as ham or bacon). Red meat included due to clear link between red meat and mortality.3	≤3 portion/week	>3 portion/week
**9. Processed meat intake**	"How often do you eat processed meats (such as bacon, ham, sausages, meat pies, kebabs, burgers, chicken nuggets)?"	≤1 portion/week	>1 portion/week

Participants were classified as "not at risk/healthy" or "at risk/unhealthy" based on their responses to questions for each lifestyle factor. *UK Biobank physical activity (PA) data were analyzed in accordance with the International Physical Activity Questionnaire (IPAQ) scoring protocol, with total physical activity computed as the sum of walking, moderate, and vigorous activity, measured as metabolic equivalents (MET-hours/week). ǂ UK Biobank participants were asked about work-related sitting time and were not asked about discretionary/leisure time sedentary behavior. Therefore, TV viewing time, which is strongly associated with mortality, was used instead of sedentary time. UK Biobank participants were asked separately about time spent using a computer, excluding computer time at work. This information was used to check for implausible values for TV viewing time. Φ National dietary guidelines were used as the basis for the dietary components.

(Developed by Hamish M, Lancet Public Health 2018)

ICD-10 codes for defining cardiovascular and thromboembolic events and diabetes.

Clinical events	ICD-10 codes
Atrial fibrillation	I48
Coronary artery disease	I21, I22, I23, I24.1, I25.2
Ischaemic stroke	I63, I64
Venous thromboembolic disease	I81, I82, I26, O22.3, O87.1, O08.2
Diabetes (the negative control outcome)	E119
